# Emerging Technologies and Generic Assays for the Detection of Anti-Drug Antibodies

**DOI:** 10.1155/2016/6262383

**Published:** 2016-07-31

**Authors:** Michael A. Partridge, Shobha Purushothama, Chinnasamy Elango, Yanmei Lu

**Affiliations:** ^1^Regeneron Pharmaceuticals, Inc., 777 Old Saw Mill River Road, Tarrytown, NY 10591, USA; ^2^UCB Pharma, Slough, Berkshire SL1 14EN, UK; ^3^Department of Biochemical & Cellular Pharmacology, Genentech Inc., South San Francisco, CA 94080, USA

## Abstract

Anti-drug antibodies induced by biologic therapeutics often impact drug pharmacokinetics, pharmacodynamics response, clinical efficacy, and patient safety. It is critical to assess the immunogenicity risk of potential biotherapeutics in producing neutralizing and nonneutralizing anti-drug antibodies, especially in clinical phases of drug development. Different assay methodologies have been used to detect all anti-drug antibodies, including ELISA, radioimmunoassay, surface plasmon resonance, and electrochemiluminescence-based technologies. The most commonly used method is a bridging assay, performed in an ELISA or on the Meso Scale Discovery platform. In this report, we aim to review the emerging new assay technologies that can complement or address challenges associated with the bridging assay format in screening and confirmation of ADAs. We also summarize generic anti-drug antibody assays that do not require drug-specific reagents for nonclinical studies. These generic assays significantly reduce assay development efforts and, therefore, shorten the assay readiness timeline.

## 1. Introduction

Biotherapeutics often elicit unwanted immune response that produces nonneutralizing and/or neutralizing anti-drug antibodies (ADAs). A widely adopted tiered immunogenicity testing approach includes first the screening and confirmation of both types of ADAs using immunoassays. Testing for neutralizing antibodies, preferably using methods that reflect the drug's mechanism of action* in vivo*, may be followed up for the ADA positive samples [1]. The industry standard for immunogenicity testing of ADA screening and confirmation is the bridging immunoassay. In these assays the drug is labeled separately with different haptens or tags and any anti-drug antibodies present in a sample will form a bridge between the two labeled molecules. The most important advantage with this method is that all isotypes of ADA (IgG, IgM, IgA, etc.) can be detected [2]. This format can also be used in all species, as any immunoglobulin is capable of forming an immune complex with the two labeled drugs.

However, there are several significant disadvantages with the bridging assay format. Bridging assays can also detect preexisting antibodies that recognize the drug. Examples of such antibodies include agents found in particular diseased populations, such as rheumatoid factor that binds to Fc domains of immunoglobulins [3–5]. In other cases preexisting antibodies reactive to specific regions, such as anti-allotype [6] or anti-hinge region [7, 8] antibodies, were detected in bridging assays. In one case, preexisting IgE antibodies specific to an oligosaccharide present on the therapeutic have also been shown to cause a hypersensitivity reaction [9]. However, preexisting reactivity has generally not been shown to be a risk factor for posttreatment immunogenicity, especially for monoclonal antibody drugs, and in most cases the agent responsible is not identified [4, 10]. Minimizing this type of interference is a challenge in bridging assays and can confound the detection and interpretation of treatment-induced ADA.

In addition to immunoglobulins, other serum components can generate signal in the assay. Soluble, multimeric drug targets can also form a bridge between the two labeled molecules, generating a false positive response [11, 12]. This is a particularly challenging problem, as soluble target levels generally increase after dosing with the drug, in some cases by substantial amounts [13], and the target-mediated assay signal needs to be reduced to below the assay cut point.

Although the bridging assay format allows detection of all isotypes of ADA, it does not identify the specific isotypes represented in the response. In addition, the bridging assay does not detect IgG4 subclass when monovalent due to Fab arm exchange [14], and the ADA response to biotherapeutics can include a substantial proportion of IgG4 [15–17]. However, in these cases there was also a significant IgG1 response. Hence, the bridging assay may underestimate the level of IgG4 ADA but would likely not generate a false negative assay response.

Drug interference is a major challenge in immunogenicity assays because the presence of drug may result in false negatives, which in turn underestimates the ADA incidence. Given these difficulties with the bridging assay format and drug tolerance issue in general, alternative platforms or strategies may be required for immunogenicity testing. The focus of this paper is to review some of the new technologies that address the challenges of bridging ELISA/MSD or complement bridging assays for the screening and confirmation of ADAs. These technologies allow identification of the specific isotypes and subclasses of ADA and can substantially reduce interference from soluble drug target. In addition, they may provide improved drug tolerance, low sample or reagent consumption, and better matrix tolerance. It is important to recognize that the ADAs detected by immunoassays may not necessarily be clinically relevant. The correlations of ADAs with clinical impact on safety, pharmacokinetics, pharmacodynamics, and efficacy can only be drawn when sufficient data has been accumulated during late stage of clinical development or even postauthorization clinical practice [18]. Finally, generic ADA assay methodologies that provide significant time saving in assay development are also discussed. Neutralizing antibody assays have been reviewed by others [1] and are outside of the scope of this paper.

## 2. Emerging Technologies for ADA Assays

### 2.1. Immunogenicity Assays Using Gyrolab

The Gyrolab platform, which incorporates microfluidics and partial automation, has been widely used as a bioanalytical tool in ligand binding assays [19]. Assays have been validated on the Gyrolab platform for quantification of biologic drug candidates (including human IgGs) to determine their pharmacokinetic properties [20, 21] and to measure the affinity of protein-protein interactions [22].

Recent industry meetings have also showcased bridging ADA assays being developed using the Gyrolab. The platform offers an ADA mixing CD where the key steps of an ADA assay, such as acid treatment, neutralization, and bridging reaction, are automated and performed within each CD microstructure [http://www.gyros.com/products/gyrolab-cds/]. Alternatively, since the technology is based on streptavidin coated beads [23], the sample preparation steps mentioned above can also be performed outside of the system [24] (similar to the MSD platform), followed by analysis on a regular Gyros CD (1000 nL or 200 nL). The Gyrolab ADA software also provides a statistical tool that can assist in cut point determination.

Among the advantages with the Gyrolab platform is the minimal use of critical reagents and sample volume due to the microfluidics [22]. For ADA assays using the ADA mixing CD, the run time per CD can be less than one hour (enabled by automating the acid and neutralization steps within the ADA CD). However, the relatively short incubation time for neutralization and bridging reaction in the ADA CD may not be sufficient for low affinity antibodies present in the samples to form the bridged complex. This can be addressed during development by optimizing the incubation times for the acid and neutralization/bridging steps.

Some of the disadvantages of the ADA CD include fewer sample throughputs per CD, 48 microstructures per CD compared to 96 or 112 (1000 or 200 nL CD used for PK assays). Also, the ADA CD has a maximum sample volume of 200 nL, which is 5 times less than the regular 1000 nL CD. Additional limitations to the Gyros platform include the cost of reagents and consumables, in particular the CDs. However, overall Gyros can be considered as an alternative platform for the development of ADA assays to improve workflow through automation or save on volume of critical reagents consumed.

### 2.2. Immunogenicity Assays Using iPCR

One of the key challenges reported for accepted methodologies for ADA testing is the interference from circulating drug. Several methodologies, prominent amongst them acid dissociation and ADA enrichment, have been reported for improving drug tolerance [25]. In addition, collecting samples after a wash-out period is also common, although this approach may not be practical in multidose efficacy studies. One way to improve drug tolerance in ADA assays is by incorporating large dilutions to reduce the concentration of drug in the sample. Technologies like immune-PCR (iPCR) that offer high sensitivity may allow for detection of ADA in diluted samples. In two reports describing ADA assays using iPCR [26, 27], the bridging assay format was used. The divalent ADA molecule forms a bridge between drugs immobilized on the plate and biotin labeled drug. The biotin labeled complexes were detected using a proprietary Imperacer® reagent, anti-biotin antibody conjugated to DNA, which is quantified by real time PCR [26].

In the first study, using a model system of goat anti-mouse IgG as protein drug mimic and a polyclonal rabbit anti-goat IgG mimicking the ADA, the assay could tolerate drug levels 2000-fold greater than ADA levels (10 ng/mL of the ADA detected in the presence of 20 mg/mL of drug), probably due to larger initial dilution of the sample causing dissociation of ADA-drug complexes. The high sensitivity of the platform due to signal amplification allows for the detection of low ADA levels. This is higher than what has been reported using sample pretreatment and acidification [25, 28, 29]. This model system also reported sensitivity in the order of 5 pg/mL [26].

The second report using iPCR for ADA detection pertains to the development of an assay to support clinical trials of a receptor IgG1 fusion protein. Drug tolerance levels were reported as 1000-fold greater than ADA levels for the iPCR, versus 40-fold greater for the MSD platform. In addition, sensitivity (using the same positive control) was 20 ng/mL and 40 pg/mL for the MSD and iPCR platforms, respectively [27]. In addition, in this case study, using the iPCR platforms allowed an investigation of whether the inverse relationship between ADA incidence and dose administered was due to the inability of the assay to detect ADA in the presence of circulating drug or the induction of immune tolerance.

One of the advantages to using iPCR (in addition to improved drug tolerance) is that the PCR amplification enables large sample dilution, thereby reducing matrix effects [26]. While the iPCR assay platform has reported improved drug tolerance and sensitivity, the platform is dependent on proprietary reagents obtained from the vendor and is therefore less amenable to in-house method development. The need for rigorous analyst training has also been mentioned as a concern for obtaining reproducible results [27].

### 2.3. Simultaneous Detection and Isotyping of an ADA Response

#### 2.3.1. SQI SquidLite Technology

Additional characterization of the immune response may require the development of assays to isotype the ADA detected. SQI Diagnostics SquidLite technology platform offers the option of ADA detection and isotyping in the same well, potentially eliminating the need for multiple assays and reducing the amount of sample volume needed. SQI's technology prints microarray spots of the drug on an activated glass surface. This is followed sequentially by sample addition and the addition of fluorescent labeled detection antibodies [30]. Using this technology platform, users have reported improved sensitivity and drug tolerance compared to an ELISA or ECL platform [30]. The advantage of the SQI platform is the ability to multiplex when isotyping is needed and the automated system can potentially increase throughput. However, the platform uses proprietary buffers and has a substantial upfront cost in instrument setup. In addition, initial method development is performed by SQI and workup is needed by the vendor to optimize spotting of the drug on the glass surface.

#### 2.3.2. Genalyte Maverick System

Another technology platform that enables simultaneous ADA detection and isotyping of the immune response is the Genalyte Maverick. In this technology platform, anti-isotype capture probes are printed on the silicon photonic biosensor surface. Anti-drug antibody in the sample is captured via the Fc portion and the complex is detected using biotinylated drug followed by streptavidin coated beads causing a change of refractive index. The Maverick system is capable of detecting all immunoglobulin isotypes including monovalent IgG4. However, this platform requires sample pretreatment steps of acid dissociation followed by affinity capture of ADA using drug coated on 96 plates. The ADAs are eluted off the plate in low pH and neutralized before flowing over the sensor chip. Both platforms (SQI and Maverick) offer the advantage of automation. Maverick is a label-free detection that allows for real time kinetic binding results, whereas SQI is end point reading. However, unlike the SQI there is no user published literature as yet on the use of the Maverick and the currently available information is primarily provided by the vendor (http://www.genalyte.com/resources/).

#### 2.3.3. Immunocapture-LC/MS

Liquid chromatography mass spectrometry (LC/MS) is becoming an important technology for large molecule drug development. The applications of LC/MS in quantifying endogenous protein biomarkers and biotherapeutics in biological matrices are increasing. Generic LC-MS assays that measure human mAbs or Fc fusion proteins have been successfully used for PK bioanalysis [31, 32]. The use of LC-MS for immunogenicity testing has also emerged in the recent years [33, 34]. In this special issue, Chen et al. reported an immunocapture-LC/MS assay for simultaneous ADA detection and isotyping [35]. The authors identified proteolytic derived surrogate peptides that are unique to each human immunoglobulin isotype and subclass by LC/MS. Human sera containing preexisting ADA against a therapeutic protein as determined by ECL bridging assay were used to demonstrate the LC/MS ADA assay technology. To increase assay sensitivity, immunopurification was first performed on these human sera using two techniques. One method is to use biotinylated drug to enrich the ADA. The other is to spike excess drug into the sera to saturate the ADA binding sites followed by using a mouse monoclonal antibody against drug to capture the ADA-drug complex. The universal peptides specific to each isotype/subclass were semiquantitated using LC/MS and purified human Ig isotype standards. LC/MS ADA assay will become a valuable technology for immunogenicity testing given LC/MS's advantage in multiplexing capability and high specificity. However, additional improvements are needed to further reduce the endogenous Ig interference of the immunocapture-LC/MS assay, and the immunocapture step itself may lead to a higher level of interlaboratory variability. Furthermore, successful use of the platform requires strong LC/MS capabilities which may not be available in standard bioanalytical laboratories. Serial analysis by LC/MS is also generally lower throughput compared to ligand binding assays performed in a 96-well immunoassay plate. Finally, the application of LC/MS in clinical immunogenicity testing needs to be demonstrated in patient samples treated with biotherapeutics.

## 3. Generic ADA Assay Methodologies

ADA assays typically use drug-specific reagents that require substantial time and resources to prepare and characterize. Generic ADA assays that can be applied to all human monoclonal antibody therapeutics have been reported for nonclinical applications [36, 37]. These “off-the-shelf” assays require minimal assay optimization and can be used for early candidate selection studies right up to IND-enabling GLP studies.

### 3.1. Generic ADA ELISAs for Nonclinical Studies

Immune-complex assays have been used to measure immunogenicity in mouse and cynomolgus monkey studies [36, 37]. The assays used anti-human constant region antibodies to capture the drug and anti-mouse or anti-cynomolgus IgG species-specific antibodies to detect the drug-ADA complex. By preincubating the sample with excess drug, the immune-complex assay measures total ADA levels. Since the first report of the assay in 2010, the monkey ADA assay has been modified and successfully used by Carrasco-Triguero et al., for not only monovalent human IgG therapeutic antibodies, but also antibody-drug conjugates (see this special issue) [38]. The modified version uses a commercially available anti-human IgG Fc specific monoclonal antibody as coat and an anti-monkey IgG polyclonal antibody for detection. In addition, the paper also described a simple methodology for determining in study cut points. This assay format has superior drug tolerance and adequate sensitivity that detected equal or greater number of ADA positive responses compared to therapeutic-specific bridging ELISA. One of the drawbacks of the generic total ADA assay is that this assay format does not allow for mapping ADA domain specificity, which was typically done by spiking in other drug-related constructs. Another difference is that bridging ELISAs detect many different isotypes whereas the ADA-drug complex assay is limited to IgG isotype. Furthermore, the complex assay method is not likely applicable to the detection of ADA in humans dosed with human monoclonal antibodies.

### 3.2. Universal ADA ECL Immunoassays for Nonclinical Studies

To simplify ADA assay development during nonclinical studies, Bautista et al. reported a universal indirect species-specific immunoassay (UNISA) for ADA detection in three animal species: mouse, rat, and cynomolgus monkey [39]. The format of these assays is to coat the therapeutic on carbon surface plates to capture ADA, which is detected with a species-specific antibody. Unlike the immune-complex assay, this assay format allows for mapping the ADA specificity to different regions of the biotherapeutic molecule.

In a follow-up paper, Bautista et al. validated the UNISA using a single assay condition across four representative therapeutic monoclonal antibodies [40]. The paper established a universal cut point, but the suitability of the cut point would need to be assessed for each new drug candidate. The paper also demonstrated similar assay sensitivity (2–10 ng/mL) and drug tolerance (272–403 ng/mL) with the positive control and defined acceptance criteria based on the four human monoclonal antibodies tested. As with the immune-complex assays, the UNISA uses generic reagents and assay conditions which is a considerable advantage in nonclinical studies. First, the coat antibody does not require labeling and is ready to use as it is. Second, using one assay condition across different therapeutic antibodies saves the assay development time. In addition, the streamlined immunogenicity assessment strategy speeds up the assay validation. As with the immune-complex generic ADA assay, the use of specific detection antibodies precludes detection of all isotypes of ADA. In addition, the method has limited application in the clinical setting.

### 3.3. Generic Human Anti-PEG Antibody Assays Using Acoustic Membrane Microparticle Technology

Polyethylene glycol (PEG) is a synthetic polymer that has wide applications, from the cosmetics industry to the biomedical field [41–43]. However, preexisting anti-PEG antibody and antibody against PEG moiety induced by PEGylated therapeutics have been observed in animal models and humans. Preexisting anti-PEG antibodies may result in an increased incidence of immunogenicity and/or in hypersensitivity reactions when patients are treated with PEGylated biotherapeutics [42].

Current immunoassays to detect anti-PEG antibody responses have suffered from a lack of well-characterized positive controls and poor specificity due to the high background seen in these assays [44]. Recently the development of a generic anti-PEG antibody assay using custom generated anti-PEG antibody and the Acoustic Membrane Microparticle Technology (AMMP or ViBE® workstation) platform has been reported [45]. In this assay, a magnetic bead conjugated with biotin PEG and bound anti-PEG antibody is detected on a Protein A functionalized sensor. The change in mass due to the bound complex changes the frequency of an oscillating piezoelectric membrane proportional to the amount of analyte bound [46]. The AMMP assay overcomes the issue of high background seen in other anti-PEG antibody assays as only beads that have the complex of PEG-anti-PEG antibody stick to the sensor. Other serum components that may bind to Protein A sensor surface do not contribute to a significant change in mass. The anti-PEG antibody assay sensitivity with this custom positive control was 800 ng/mL on the AMMP compared to the 50–500 *μ*g/mL range seen with the MSD and AlphaLISA platforms with the same positive control [45]. The use of a Protein A surface increases the assay sensitivity owing to the multiple IgG binding domains on Protein A. However, direct binding methods also detect IgM which may be a substantial component of any anti-PEG response [42]. During method development on the AMMP platform, it is important to determine the extent of protein coverage on the bead surface, the need for additional wash steps (and their impact on the ability to detect low affinity ADA). A particularly important consideration for anti-PEG antibody assays is the removal of surfactants like Tween that have a framework structure similar to PEG.

## 4. Conclusion

The interpretation of immunogenicity data from bridging ADA assays has been widely accepted by industry and regulators for approval and marketing of biotherapeutics. Given the ubiquity of the bridging assay platform, many of the technologies discussed here have been targeted for particular uses (e.g., ADA isotyping, obtaining high sensitivity, or improving drug tolerance). However, immunogenicity data from marketed products that have been extensively studied indicate that ADA positive patients identified using assays with higher drug tolerance have a lower correlation with clinical efficacy [18]. This suggests that more sensitive and drug tolerant assays may not always help interpret the impact of ADA on clinical outcomes. Nevertheless, regulatory authorities continue to request additional details on the nature of immunogenic responses to ensure patient safety. The alternative platforms described in this review may help overcome some of the limitations of the bridging assay or provide options for further characterization of antibodies against therapeutic proteins.

## References

[1] Shankar G., Pendley C., Stein K. E. (2007). A risk-based bioanalytical strategy for the assessment of antibody immune responses against biological drugs. *Nature Biotechnology*.

[2] Mire-Sluis A. R., Barrett Y. C., Devanarayan V. (2004). Recommendations for the design and optimization of immunoassays used in the detection of host antibodies against biotechnology products. *Journal of Immunological Methods*.

[3] Myler H., Felix T., Zhu J., Hruska M., Piccoli S. P. (2014). Measuring biotherapeutics with endogenous counterparts and pre-existing antibodies: an interferon case study. *Bioanalysis*.

[4] Van Schie K. A., Wolbink G.-J., Rispens T. (2015). Cross-reactive and pre-existing antibodies to therapeutic antibodies—effects on treatment and immunogenicity. *mAbs*.

[5] Gorovits B., McNally J., Fiorotti C., Leung S. (2014). Protein-based matrix interferences in ligand-binding assays. *Bioanalysis*.

[6] Tatarewicz S. M., Juan G., Swanson S. J., Moxness M. S. (2012). Epitope characterization of pre-existing and developing antibodies to an aglycosylated monoclonal antibody therapeutic of G1m17,1 allotype. *Journal of Immunological Methods*.

[7] Ben-Horin S., Yavzori M., Katz L. (2011). The immunogenic part of infliximab is the F(ab')2, but measuring antibodies to the intact infliximab molecule is more clinically useful. *Gut*.

[8] Rispens T., De Vrieze H., De Groot E. (2012). Antibodies to constant domains of therapeutic monoclonal antibodies: anti-hinge antibodies in immunogenicity testing. *Journal of Immunological Methods*.

[9] Chung C. H., Mirakhur B., Chan E. (2008). Cetuximab-induced anaphylaxis and IgE specific for galactose-*α*-1,3- galactose. *The New England Journal of Medicine*.

[10] Xue L., Rup B. (2013). Evaluation of pre-existing antibody presence as a risk factor for posttreatment anti-drug antibody induction: analysis of human clinical study data for multiple biotherapeutics. *The AAPS Journal*.

[11] Carrasco-Triguero M., Mahood C., Milojic-Blair M. (2012). Overcoming soluble target interference in an anti-therapeutic antibody screening assay for an antibody-drug conjugate therapeutic. *Bioanalysis*.

[12] Zhong Z. D., Dinnogen S., Hokom M. (2010). Identification and inhibition of drug target interference in immunogenicity assays. *Journal of Immunological Methods*.

[13] Nishimoto N., Terao K., Mima T., Nakahara H., Takagi N., Kakehi T. (2008). Mechanisms and pathologic significances in increase in serum interleukin-6 (IL-6) and soluble IL-6 receptor after administration of an anti-IL-6 receptor antibody, tocilizumab, in patients with rheumatoid arthritis and Castleman disease. *Blood*.

[14] Hart M. H., De Vrieze H., Wouters D. (2011). Differential effect of drug interference in immunogenicity assays. *Journal of Immunological Methods*.

[15] Barger T. E., Wrona D., Goletz T. J., Mytych D. T. (2012). A detailed examination of the antibody prevalence and characteristics of anti-ESA antibodies. *Nephrology Dialysis Transplantation*.

[16] Van Schouwenburg P. A., Rispens T., Wolbink G. J. (2013). Immunogenicity of anti-TNF biologic therapies for rheumatoid arthritis. *Nature Reviews Rheumatology*.

[17] Van Schouwenburg P. A., Krieckaert C. L., Nurmohamed M. (2012). IgG4 production against adalimumab during long term treatment of RA patients. *Journal of Clinical Immunology*.

[18] Van Schouwenburg P. A., Krieckaert C. L., Rispens T., Aarden L., Wolbink G. J., Wouters D. (2013). Long-term measurement of anti-adalimumab using pH-shift-anti-idiotype antigen binding test shows predictive value and transient antibody formation. *Annals of the Rheumatic Diseases*.

[19] Eriksson C., Agaton C., Kånge R. (2006). Microfluidic analysis of antibody specificity in a compact disk format. *Journal of Proteome Research*.

[20] Magana I., Macaraeg C. R., Zhang L., Ma M., Thway T. M. (2014). Validation of a microfluidic platform to measure total therapeutic antibodies and incurred sample reanalysis performance. *Bioanalysis*.

[21] Liu X. F., Wang X., Weaver R. J. (2012). Validation of a gyrolab*™* assay for quantification of rituximab in human serum. *Journal of Pharmacological and Toxicological Methods*.

[22] Salimi-Moosavi H., Rathanaswami P., Rajendran S., Toupikov M., Hill J. (2012). Rapid affinity measurement of protein-protein interactions in a microfluidic platform. *Analytical Biochemistry*.

[23] Given A. M., Whalen P. M., O'Brien P. J., Ray C. A. (2012). Development and validation of an alpha fetoprotein immunoassay using Gyros technology. *Journal of Pharmaceutical and Biomedical Analysis*.

[24] Mikulskis A., Yeung D., Subramanyam M., Amaravadi L. (2011). Solution ELISA as a platform of choice for development of robust, drug tolerant immunogenicity assays in support of drug development. *Journal of Immunological Methods*.

[25] Smith H. W., Butterfield A., Sun D. (2007). Detection of antibodies against therapeutic proteins in the presence of residual therapeutic protein using a solid-phase extraction with acid dissociation (SPEAD) sample treatment prior to ELISA. *Regulatory Toxicology and Pharmacology*.

[26] Spengler M., Adler M., Jonas A., Niemeyer C. M. (2009). Immuno-PCR assays for immunogenicity testing. *Biochemical and Biophysical Research Communications*.

[27] Jani D., Savino E., Goyal J. (2015). Feasibility of immuno-PCR technology platforms as an ultrasensitive tool for the detection of anti-drug antibodies. *Bioanalysis*.

[28] Bourdage J. S., Cook C. A., Farrington D. L., Chain J. S., Konrad R. J. (2007). An Affinity Capture Elution (ACE) assay for detection of anti-drug antibody to monoclonal antibody therapeutics in the presence of high levels of drug. *Journal of Immunological Methods*.

[29] Sickert D., Kroeger K., Zickler C. (2008). Improvement of drug tolerance in immunogenicity testing by acid treatment on Biacore. *Journal of Immunological Methods*.

[30] Mora J., Given Chunyk A., Dysinger M. (2014). Next generation ligand binding assays—review of emerging technologies’ capabilities to enhance throughput and multiplexing. *The AAPS Journal*.

[31] Furlong M. T., Ouyang Z., Wu S. (2012). A universal surrogate peptide to enable LC-MS/MS bioanalysis of a diversity of human monoclonal antibody and human Fc-fusion protein drug candidates in pre-clinical animal studies. *Biomedical Chromatography*.

[32] Li H., Ortiz R., Tran L. (2012). General LC-MS/MS method approach to quantify therapeutic monoclonal antibodies using a common whole antibody internal standard with application to preclinical studies. *Analytical Chemistry*.

[33] Jiang H., Xu W., Titsch C. A. (2014). Innovative use of LC-MS/MS for simultaneous quantitation of neutralizing antibody, residual drug, and human immunoglobulin G in immunogenicity assay development. *Analytical Chemistry*.

[34] Neubert H., Grace C., Rumpel K., James I. (2008). Assessing immunogenicity in the presence of excess protein therapeutic using immunoprecipitation and quantitative mass spectrometry. *Analytical Chemistry*.

[35] Chen L.-Z., Roos D., Philip E. (2016). Development of immunocapture-LC/MS assay for simultaneous ADA isotyping and semiquantitation. *Journal of Immunology Research*.

[36] Stubenrauch K., MacKeben K., Vogel R., Heinrich J. (2012). Generic anti-drug antibody assay with drug tolerance in serum samples from mice exposed to human antibodies. *Analytical Biochemistry*.

[37] Stubenrauch K., Wessels U., Essig U., Vogel R., Schleypen J. (2010). Evaluation of a generic immunoassay with drug tolerance to detect immune complexes in serum samples from cynomolgus monkeys after administration of human antibodies. *Journal of Pharmaceutical and Biomedical Analysis*.

[38] Carrasco-Triguero M., Davis H., Zhu Y. (2016). Application of a plug-and-play immunogenicity assay in cynomolgus monkey serum for ADCs at early stages of drug development. *Journal of Immunology Research*.

[39] Bautista A. C., Salimi-Moosavi H., Jawa V. (2012). Universal immunoassay applied during early development of large molecules to understand impact of immunogenicity on biotherapeutic exposure. *The AAPS Journal*.

[40] Bautista A. C., Zhou L., Jawa V. (2013). Universal immunogenicity validation and assessment during early biotherapeutic development to support a green laboratory. *Bioanalysis*.

[41] Hu X., Miller L., Richman S. (2012). A novel PEGylated interferon beta-1a for multiple sclerosis: safety, pharmacology, and biology. *Journal of Clinical Pharmacology*.

[42] Myler H., Hruska M. W., Srinivasan S. (2015). Anti-PEG antibody bioanalysis: a clinical case study with PEG-IFN-*λ*-1a and PEG-IFN-*α*2a in naive patients. *Bioanalysis*.

[43] White J. T., Crossman M., Richter K., Berman M., Goyal J., Subramanyam M. (2015). Immunogenicity evaluation strategy for a second-generation therapeutic, PEG-IFN-*β*-1a. *Bioanalysis*.

[44] Schellekens H., Hennink W. E., Brinks V. (2013). The immunogenicity of polyethylene glycol: facts and fiction. *Pharmaceutical Research*.

[45] Dong H., Mora J. R., Brockus C. (2015). Development of a generic anti-PEG antibody assay using bioscale’s acoustic membrane microparticle technology. *The AAPS Journal*.

[46] Chilewski S. D., Dickerson W. M., Mora J. R., Saab A., Alderman E. M. (2014). Evaluation of acoustic membrane microparticle (Ammp) technology for a sensitive ligand binding assay to support pharmacokinetic determinations of a biotherapeutic. *The AAPS Journal*.

